# KCTD10 Is Involved in the Cardiovascular System and Notch Signaling during Early Embryonic Development

**DOI:** 10.1371/journal.pone.0112275

**Published:** 2014-11-17

**Authors:** Kaiqun Ren, Jing Yuan, Manjun Yang, Xiang Gao, Xiaofeng Ding, Jianlin Zhou, Xingwang Hu, Jianguo Cao, Xiyun Deng, Shuanglin Xiang, Jian Zhang

**Affiliations:** 1 Key Laboratory of Protein Chemistry and Developmental Biology of State Education Ministry of China, College of Life Science, Hunan Normal University, Changsha, P. R. China; 2 Model Animal Research Center, MOE Key Laboratory of Model Animal for Disease Research, Medical School, Nanjing University, Nanjing, P.R. China; 3 College of Medicine, Hunan Normal University, Changsha, P. R. China; Northwestern University, United States of America

## Abstract

As a member of the polymerase delta-interacting protein 1 (PDIP1) gene family, potassium channel tetramerisation domain-containing 10 (KCTD10) interacts with proliferating cell nuclear antigen (PCNA) and polymerase δ, participates in DNA repair, DNA replication and cell-cycle control. In order to further investigate the physiological functions of KCTD10, we generated the KCTD10 knockout mice. The heterozygous KCTD10^+/−^ mice were viable and fertile, while the homozygous KCTD10^−/−^ mice showed delayed growth from E9.0, and died at approximately E10.5, which displayed severe defects in angiogenesis and heart development. Further study showed that VEGF induced the expression of KCTD10 in a time- and dose-dependent manner. Quantitative real-time PCR and western blotting results revealed that several key members in Notch signaling were up-regulated either in KCTD10-deficient embryos or in KCTD10-silenced HUVECs. Meanwhile, the endogenous immunoprecipitation (IP) analysis showed that KCTD10 interacted with Cullin3 and Notch1 simultaneously, by which mediating Notch1 proteolytic degradation. Our studies suggest that KCTD10 plays crucial roles in embryonic angiogenesis and heart development in mammalians by negatively regulating the Notch signaling pathway.

## Introduction

KCTD10 is a member of the polymerase delta-interacting protein 1 (PDIP1) gene family [1], which consists of 3 members, PDIP1, KCTD10 and TNFAIP1 [2]–[5]. All the three members contain a conserved BTB/POZ domain, a potassium channel tetramerisation (K-tetra) domain (a relative of BTB/POZ domain) at the N-terminus, and a proliferating cell nuclear antigen (PCNA)-binding motif at the C-terminus [1], [4]. KCTD10 is inducible by TNF-α, interacts with PCNA and the small subunit (p50) of DNA polymerase δ [3]. In A549 lung adenocarcinama cells, knockdown of KCTD10 decreases PCNA expression [6]. Promoter analysis showed that KCTD10 can be regulated positively by SP1 and negatively by AP-2 transcription factors [5]. In a recent study, KCTD10 was reported to be regulated by a novel transcription factor ETV1 which is unique to gastrointestinal stromal tumors (GISTs), and RNAi-mediated silencing of KCTD10 increased cell invasion, suggesting that KCTD10 function as a tumor suppressor protein [7]. However, the exact functions of KCTD10 in mammalian development remain unclear. Reports showed that KCTD10 was highly expressed in human heart, skeletal muscle, and placenta, and may regulate the development of neural tube, neuroepithelium and the dorsal root ganglion in mammals [8], suggesting that this protein may play important roles in tissue development [3], [6].

Formation of the vascular system is one of the earliest and most important events during embryogenesis in mammals. Among the early stages of vascular development in both the mammalian embryo and its extra-embryonic membranes, endothelial cell precursors differentiate and coalesce into a network of homogeneously sized primitive blood vessels in a process termed vasculogenesis [9]. This primary vascular plexus is then remodeled by the process of angiogenesis, which involves the sprouting, branching, splitting, and differential growth of vessels in the primary plexus to form both the large and small vessels of the mature vascular system [9], [10]. In the mesoderm, cardiovascular system arises, pluripotent hemangioblast cells give rise to the blood islands, meanwhile the peripheral cells differentiate into endothelial cells (ECs) which later form the capillaries [11]. The vasculogenesis and angiogenesis begin at E8.5 in mouse embryonic development. As blood vessels are essential for the transport of fluids, gases, nutrients, and signaling molecules between placenta and embryos, many genes’ mutation related to angiogenesis causes embryonic delay or embryonic lethality between E8.5 and E11.5.

One of the most important pathways that control the vascular differentiation is Notch signaling, which is critically involved in many cellular processes including cell proliferation, survival, apoptosis, migration, invasion, angiogenesis, and metastasis [12]. Functional studies showed that Notch signaling is crucial for the angiogenic growth in mice, fish, and human. In mice, the absence of Notch signaling results in defective yolk sac vascular remodeling and aberrant formation of arterial-venous circuits in the embryos, that often leads to embryonic death [13]. There are four different Notch receptors in mammals, referred to as Notch1, Notch2, Notch3, and Notch4. Both Notch1 and Notch4 display prominent arterial expression [13]. Mice with a dual Notch1 and Notch4 deletion show severe defects in angiogenesis: the uniform vessel networks initially form in the yolk sac but fail to properly remodel into large vessels and small capillaries [14], [15]. Notch2 is highly expressed in the heart myocardium [16]. Mice homozygous for the Notch2 mutation died perinatally from defects in heart development including pericardial edema and myocardial wall atrophy [17]. Notch3 is associated with CADASIL (Cerebral Autosomal Dominant Arteriopathy with Subcortical Infarcts and Leukoencephalopathy), which is a rare autosomal dominant genetic disease characterized by recurrent stroke, migraine headaches, cognitive deficits, and psychiatric symptoms [18]. In mammals, Hey and Hes represent the main Notch signal transducers during development [19]. The combined loss of Hey1 and Hey2 leads to a lethality vascular defect that affects the placenta, yolk sac, and embryo itself, which has been attributed to impaired arterial fate determination and maturation [20].

In this study, we found the KCTD10-deficient mouse embryos showed delayed growth from E9.0, and died at approximately E10.5 due to angiogenesis defects, heart and neuron developmental failure. Further research showed that the key members in Notch signaling, such as Dll4, Notch1 and Notch4 were up-regulated in KCTD10-deficient mice. Molecular biology studies showed that KCTD10 negatively regulated Notch signaling by mediating Notch1 proteolytic degradation.

## Materials and Methods

### Mice

Mice of C57BL/6J, 129P strains were obtained from the Jackson Laboratory (Bar Harbor, ME). Mice were maintained on a normal 12 h/12 h light/dark cycle with regular mouse chow and water ad libitum at an AAALAC accredited specific pathogen-free facility. Animal welfare and experimental procedures were carried out strictly in accordance with the care and use of laboratory animals (National Research Council, 1996). All the animals were well regulated and animal ethics were approved in this research. All animal experiments were performed in accordance with the institutional guidelines of the Model Animal Research Center, Nanjing University, and Hunan Normal University. The University Committee on Animal Care of Nanjing University and Hunan Normal University approved the experimental protocols.

### Generation of KCTD10^−/−^ mice

We used the standard BAC (Bacterial Artificial Clone) retrieval method to construct the *Kctd10* flox allele [21]. Briefly, a 9.6-kb DNA fragment containing the targeting region was retrieved from the BAC containing the whole genomic DNA sequence of *Kctd10*. The first loxP site was inserted into intron 2, and the second loxP together with neomycin-resistant gene flanked by FRT sites were inserted into intron 3. The construct was electroporated into 129 derived R1 embryonic stem (ES) cells. [22]. Targeted ES cell clones were identified by PCR and southern blotting. Chimeric mice were generated by microinjection of the positive ES cells into C57BL/6J strain blastocysts. Genetic transmission was confirmed by backcrossing the chimera to C57BL/6J mice. As previously described [23], the Neo-cassette was removed by mating to FLP-ER transgenic mice (129S4/SvJaeSor-Gt(ROSA)26Sortm1(FLP1)Dym/J, the Jackson Laboratory). To get the allele deletion of *Kctd10*, the floxed mice were crossed to EIIA-Cre (FVB/N-Tg(EIIa-cre)C5379Lmgd/J) transgenic mice (The Jackson Laboratory, Bar Harbor, ME, stock number #003314) [24] to remove the genomic DNA fragment between the two loxP sites that includes the 2^nd^ exon of *Kctd10*. Then we back-crossed the positive pups onto C57BL/6J mice to obtain heterozygous KCTD10^+/−^ mice. The homozygous KCTD10^−/−^ embryos were obtained by inter-cross of KCTD10^+/−^. The genotyping of the pups were identified by PCR analysis, all the genotyping primers and estimated size of PCR products are shown in Table 1.

**Table 1:** Oligonucleotide primers used in this study and estimated size of PCR products.

Name of primers	Sequences (5′to 3′)
KCTD10 genotyping P1	TATCTATGTCCTGTATTGTACCAG
KCTD10 genotyping P2	CAGGAGCGGAAGATAACACCAAA
KCTD10 genotyping P3	CGGGAGTGTAGGAACTAGGCTGAA
KCTD10 5-prob-F	CTGCATTGAGCGAGCTGGGTGTT
KCTD10 5-prob-R	CAGACTTTGCTCGATTCCAAGGGTA
KCTD10 3-prob-F	GAAGCCCGCATTTATGAGGAGAC
KCTD10 3-prob-R	CCAACTGCCAAACTAAGTCCTTGA
KCTD10 cDNA clone sense primer	CTCGGAATTCCGATGGAAGAGATGTCAGGAGAC
KCTD10 cDNA clone anti-sense primer R	CTGAGAATTCTCACTGGTGGAGGTGGGC
KCTD10 siRNA sequence	CCAGCAAUUCUGACGACAATTUUGUCGUCAGAAUUGCUGGTA
KCTD10 siRNA NC sequence	GGGCCGGAAGAUUGCUGAATTUUCAGCAAUCUUCCGGCCCTG
KCTD10 sense for IHC	GCAACTGAGTCCAGCTAGGG
KCTD10 antisense for IHC	TGTGAGCCCTTAGTGTGCAG
Dll4 sense	GGAACCTTCTCACTCAACATCC
Dll4 antisense	CTCGTCTGTTCGCCAAATCT
JAG1 sense	TCTCTGACCCCTGCCATAAC
JAG1 antisense	TTGAATCCATTCACCAGATCC
Mfng sense	CACCCTCAGCTACGGTGTCT
Mfng antisense	GGGTGTGCTGGGTAGAGGA
Hes1 sense	ACACCGGACAAACCAAAGAC
Hes1 antisense	CGCCTGTTCTCCATGATAGG
Hey1 sense	CATGAAGAGAGCTCACCCAGA
Hey1 antisense	CGCCGAACTCAAGTTTCC
GAPDH sense	ACCACAGTCCATGCCATCAC
GAPDH antisense	TCCACCACCCTGTTGCTGTA
KCTD10 siRNA	CCAGCAAUUCUGACGACAATTUUGUCGUCAGAAUUGCUGGTA
KCTD10 nc siRNA	CCGUGAAGUUGCUCUACAATTUUGUAGAGCAACUUCACGGCT
Name of primers	Estimated size of PCR products
KCTD10 genotyping P1	WT allele: 4 kb
KCTD10 genotyping P2	KCTD10^+/−^: 670 bp+306 bp
KCTD10 genotyping P3	KCTD10^−/−^: 306 bp

The oligonucleotide sequences used in this study are listed in the upper table. The oligonucleotide primers were used to genotype the mice, to obtain genomic fragments of *Kctd10*. The estimated sizes of PCR products obtained during genotyping the mice are indicated in the lower table.

### Plasmid construction

We amplified the coding sequence of mouse KCTD10 cDNA (GenBank Accession No. NM_026145) from mouse brain cDNA library using indicated primers as shown in Table 1. HA-KCTD10 was generated by inserting the above mouse KCTD10 cDNA into the plasmid pCMV-HA (Clontech). For KCTD10 RNA probe synthesis that used in situ hybridization, the probe fragment was digested by EcoR I from the plasmid HA-KCTD10 and inserted into the pBluescript II SK vector. Positive clones were verified by restriction enzyme digestion and sequencing. HA-cullin3 was kindly provided by Dr. Yue Xiong (University of North Carolina at Chapel Hill).

### Cell culture, siRNA transfection, VEGF induction and western blotting

HUVECs (human umbilical vessel endothelial cells, Clonetics, Inc.) were grown in modified MCDB 131 medium supplemented with 12 mg/mL bovine brain extracts (BBE), 0.01 mg/mL human epidermal growth factor (hEGF), 1 mg/mL hydrocortisone, 2% FBS and 50 mg/mL gentamycin (as recommended by the HUVEC culture protocol of Clonetics). Cells were transfected with KCTD10 siRNA or negative control siRNA as detailed in Table 1 by Lipofectamine 2000 (Invitrogen) according to the manufacturer’s instructions. Cells were harvested 24 h post-transfection.

HUVECs were treated with 10 ng/mL VEGF-A_165_ (Sigma) for the indicated time (0, 30, 45, 60, 90 and 120 min), or treated with different concentrations (0, 5, 10 ng/mL). After treatment, the cells were harvested and lysed in RIPA buffer (50 mM Tris-HCl (pH 7.2), 150 mM NaCl, 1% (v/v) Triton X-100, 1% (w/v) sodium deoxycholate, 0.1% (w/v) SDS and protease inhibitors) for protein extraction. Sample proteins were separated on 10% SDS-PAGE gel and transferred onto a PVDF membrane (Bio-Rad, Richmond, CA). Then the membrane was detected by rabbit polyclonal anti- KCTD10 (Nanjing Chuanbo Biotech Co., Ltd.), anti-Notch1 (Santa Cruze, sc-9170), anti-Notch4 (Santa Cruze, sc-5594), anti-Jag1 (Santa Cruze, sc-8303), or anti-Fringe (Santa Cruze, sc-100756), anti-NICD (CST, 2421S) antibodies separately.

### Semiquantitative and real-time RT-PCR

Total RNAs from embryos at different points were isolated using Trizol reagent (Invitrogen, Carlsbad, CA). Subsequently, the first cDNA strand was synthesized according to the manufacturer’s protocol (Qiagen, Valencia, CA). The mRNA levels of KCTD10, Dll4, JAG1, Mfng, Hes1, Hey1 and GAPDH were quantified using TaqMan RT-PCR on the ABI Prism 7700 sequence detection platform. Cycle threshold (Ct) was determined in the exponential phase of the amplification curve. Human GAPDH was used as the internal control. The ΔΔCt method was used to calculate fold changes in mRNA levels between controls and treated samples.

### Histology and immunostaining

Embryos were fixed in 4% paraformaldehyde (Sigma) and embedded in paraffin (Sigma). Then the embryos were cut into sections of 6-µm thickness, and stained with hematoxylin and eosin (H&E, Sigma) or analyzed by immunohistochemistry using polyclonal goat anti-mouse KCTD10 antibody according to the manufacturer’s instructions.

For whole-mount staining of blood vessels, embryos or yolk sacs were fixed in 4% paraformaldehyde, then blocked in PBS containing 3% milk and 0.3% triton X-100, and incubated with rat monoclonal antibody against mouse PECAM-1 (BD Pharmingen) overnight at 4°C. Alexa 594-conjugated antibody (Molecular Probes, Eugene, OR) was used as a secondary antibody. After intermittent washing, the samples were mounted and analyzed under a Leica DMZRB microscope.

For staining of cryosections, tissues were fixed in 4% paraformaldehyde for 2 h on ice, incubated in 20% sucrose/PBS overnight and embedded in O.C.T. compound (Tissue-Tek, Sakura Finetek USA, Inc., Torrance, CA). Sections were then incubated with primary antibodies that diluted in PBS with 1% (v/v) normal goat serum for 1 h and with the secondary antibodies under the same conditions. The primary antibodies used were anti-KCTD10 or anti-PECAM-1antibodies as mentioned above, while the secondary antibodies were Alexa 594 goat anti-rat and Alexa 488 goat anti-Rabbit antibodies (Molecular Probes). Hoechst 33258 (Sigma) was used to stain the nucleus. The slides were mounted with 50% glycerol in PBS and images were acquired under a fluorescence microscope (Leica).

### Whole mount in situ hybridization

Embryos were fixed and processed according to the previously published protocols [25] with the following modifications: Endogenous peroxidases were quenched with 6% H_2_O_2_ for 2 h prior to proteinase K digestion and hybridization. Hybridization was performed for 40 h at 63°C in 5× SSC (pH 4.5), 50% formamide, 5 mM EDTA, 50 µg/mL yeast tRNA, 0.2% Tween 20, 0.5% CHAPS, and 100 µg/mL heparin. Color was developed with the NBT/BCIP substrate. Embryos were post-fixed and photographed in 50% glycerol in PBS.

## Results

### KCTD10 is expressed during the early phase of mouse development

**Figure 1:**
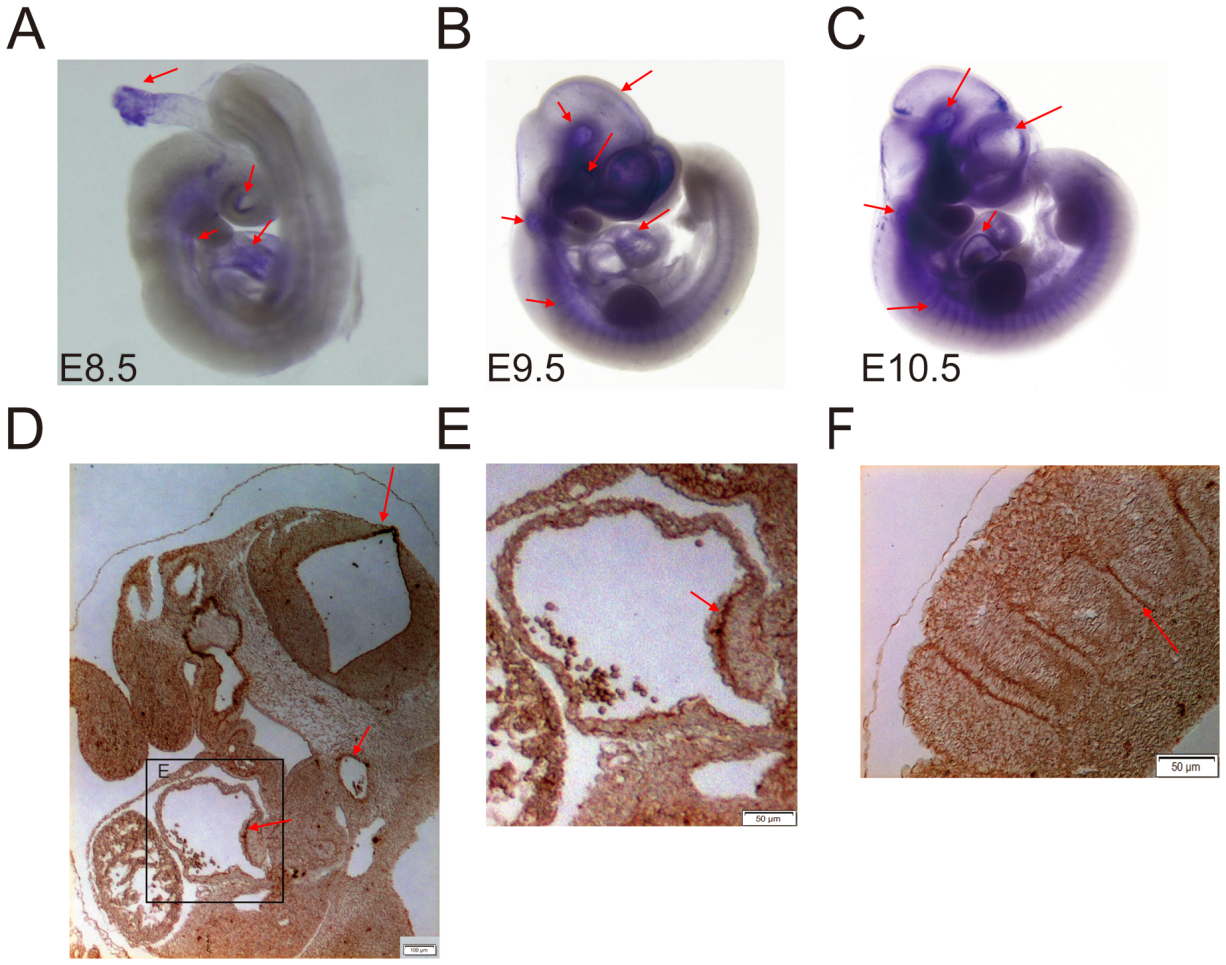
KCTD10 is expressed during the early phase of mouse development. (A–C), whole mount in situ hybridization analysis indicates KCTD10 is mainly expressed in the heart, brain, dorsal aorta and umbilical vein during the early stages of mouse development. (D–F), immunohistochemistry of paraffin sectioned E9.5 embryos exhibited strong KCTD10 mRNA signals in the neuroepithelium of the brain, dorsal aorta, heart and the boundaries of somites.

It was previously demonstrated that KCTD10 is predominately expressed in the lung, followed by the heart and testis in the rat [3]. Other groups cloned KCTD10 from a human aorta cDNA library, and their data showed a high level of KCTD10 expression in adult human heart, skeletal muscle and placenta [6]. In order to explore the functions of KCTD10 during mouse early embryogenesis, whole-mount in situ hybridization was used to determine the temporal expression of KCTD10. Embryos were collected and stained from embryonic day of 8.5. At E8.5, when embryos completed the “turning” process that included development of onset of heart and blood vessels, KCTD10 mRNA signals mainly appeared in the heart, brain, dorsal aorta and umbilical vein (Fig. 1A). While at E9.5 and E10.5, strong KCTD10 signals were detected in the brain neuroepithelium, the optic vessel, otic vesicle, only weak signals were observed in the heart and the dorsal aorta (DA) (Figs. 1B, C). In addition, sections of the brain neuroepithelium, dorsal aorta and heart of E9.5 embryos showed KCTD10 mRNA signals (Figs. 1D, E). In addition, in situ hybridization and immunohistochemistry results indicated that KCTD10 was also expressed in the somite boundaries (Figs. 1B, C and F). Thus, mouse KCTD10 was mainly expressed in the dorsal aorta and heart at E8.5, and strongly expressed in brain neuroepithelium, optic vessel, and otic vesicle at E9.5 in embryos. These results indicate that KCTD10 might play crucial roles during the mouse angiogenesis and neurogenesis in early embryogenesis.

### Deletion of KCTD10 is associated with embryonic lethality and growth retardation

**Figure 2:**
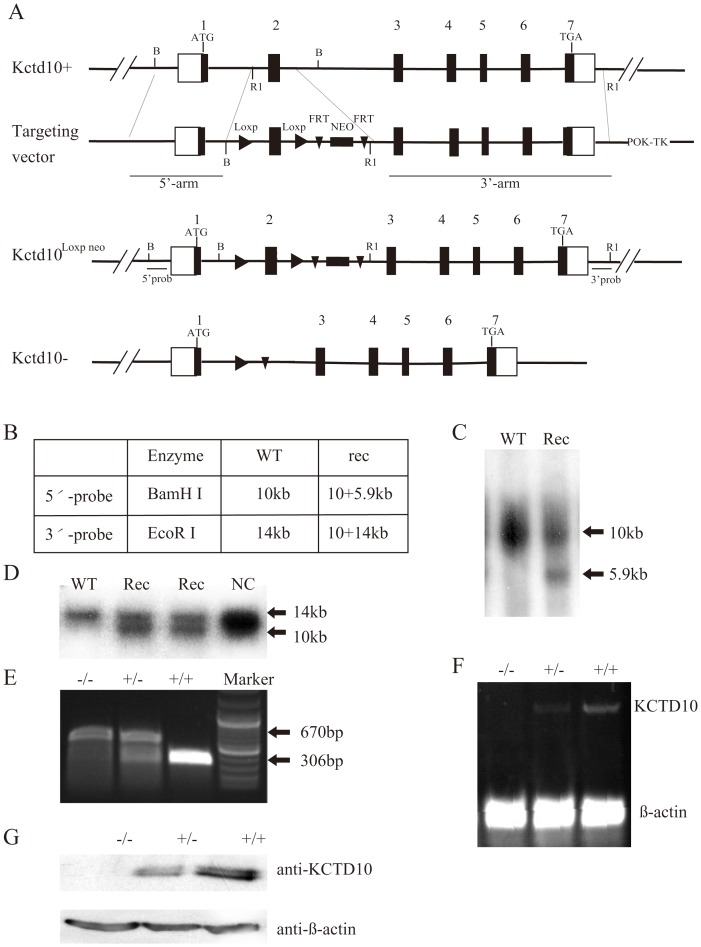
Generation of KCTD10^−/−^ mice. (A), the genomic DNA of *Kctd10* gene includes 7 exons and 6 introns. The genetic manipulation of *Kctd10* was designed to delete exon 2, the first loxP was inserted into intron 2, and the second loxP together with neomycin-resistant gene flanked by FRT sites was inserted into intron 3. (B–D), Gene disruption was confirmed using Southern blotting in positive ES cells. (E), Genotyping strategy of the mutant mouse, there is only a 670 bp band in KCTD10^−/−^ mice, two bands (670 bp and 306 bp) in the KCTD10^+/−^ mice, and only a 306 bp in the wild type mice. (F–G), RT-PCR and western blotting results indicate that the deletion is successful. (F), embryos with the same genotype were collected, and total RNA was extracted separately, reverse transcripted into cDNA and real-time PCR was performed to determine the KCTD10 mRNA levels. (G), embryos with the same genotype were collected, and total protein was extracted separately and immunoblotted by anti-KCTD10 antibody.

The gene *Kctd10* consists of seven exons, and encodes a 35 kD protein. The *Kctd10* targeting constructs were designed to delete endogenous exon 2, which resulted in KCTD10 loss of function of KCTD10 (Fig. 2A). The positive ES cells and knockout mice were confirmed by Southern blotting (Figs. 2B, C and D), genotyping (Fig. 2E), RT-PCR (Fig. 2F) and western blotting (Fig. 2G) analysis. The heterozygous KCTD10^+/−^ mice were viable and fertile, but there were no homozygous KCTD10^−/−^ mice among the off-springs. We dissected the embryos from E8.5 to E14.5, and found no detectable differences between littermates at E8.5. While at E9.5, some embryos showed developmental delay. Genotyping analysis revealed that these embryos were homozygous KCTD10^−/−^ mice. Further analysis indicated that the mutant embryos showed developmental delay from E9.5, exhibited severe morphological abnormalities, and died between E10.5 and E11.5. The ratio of mutants was about 25% (Table 2), which is consistent with the Mendel’s principles of inheritance.

**Table 2:** statistical of abnormal embryos at E9.5–E14.5.

	Normal	Abnormal	Ratio
E9.5–E10.5	156/197	41/197	20.81%
	**Normal**	**Dead**	**Ratio**
E11.5–E12.5	57/73	16/73	21.92%
	**Normal**	**Reabsorbed**	**Ratio**
E13.5–E14.5	55/70	15/70	27.27%
	**Normal**	**Abnormal**	**Ratio**
Total	268/340	72/340	21.18%

### KCTD10 deficient embryos show angiogenic defects

**Figure 3:**
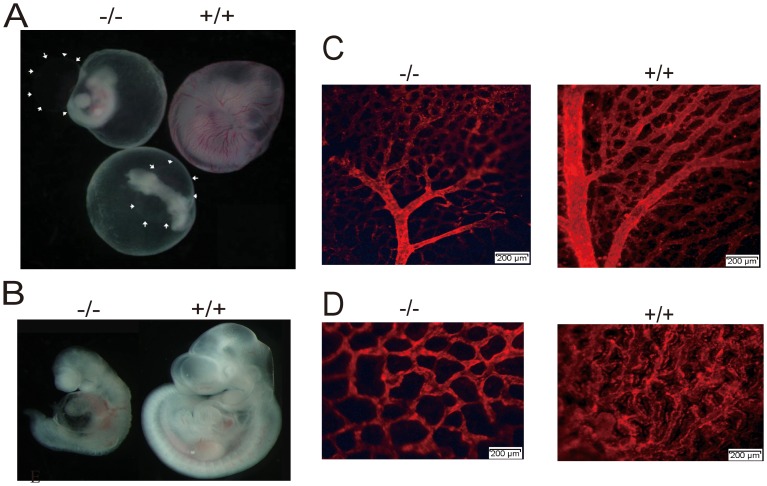
KCTD10-deficient embryos show angiogenesis defects. (A), embryos at E9.5 were dissected, and were taken photos under the stereomicroscope before the yolk sacs were split. The white arrowhead indicates the enlarged pericardial space. (B), embryos of (A) were been split the yolk sac and taken photos under the stereomicroscope, showing the underdeveloped of KCTD10^−/−^ mice. (C, D), PECAM-1 immunostaining of the yolk sacs, showing angiogenesis defects(C) and tip cells (D) in KCTD10^−/−^ mice.

**Figure 4:**
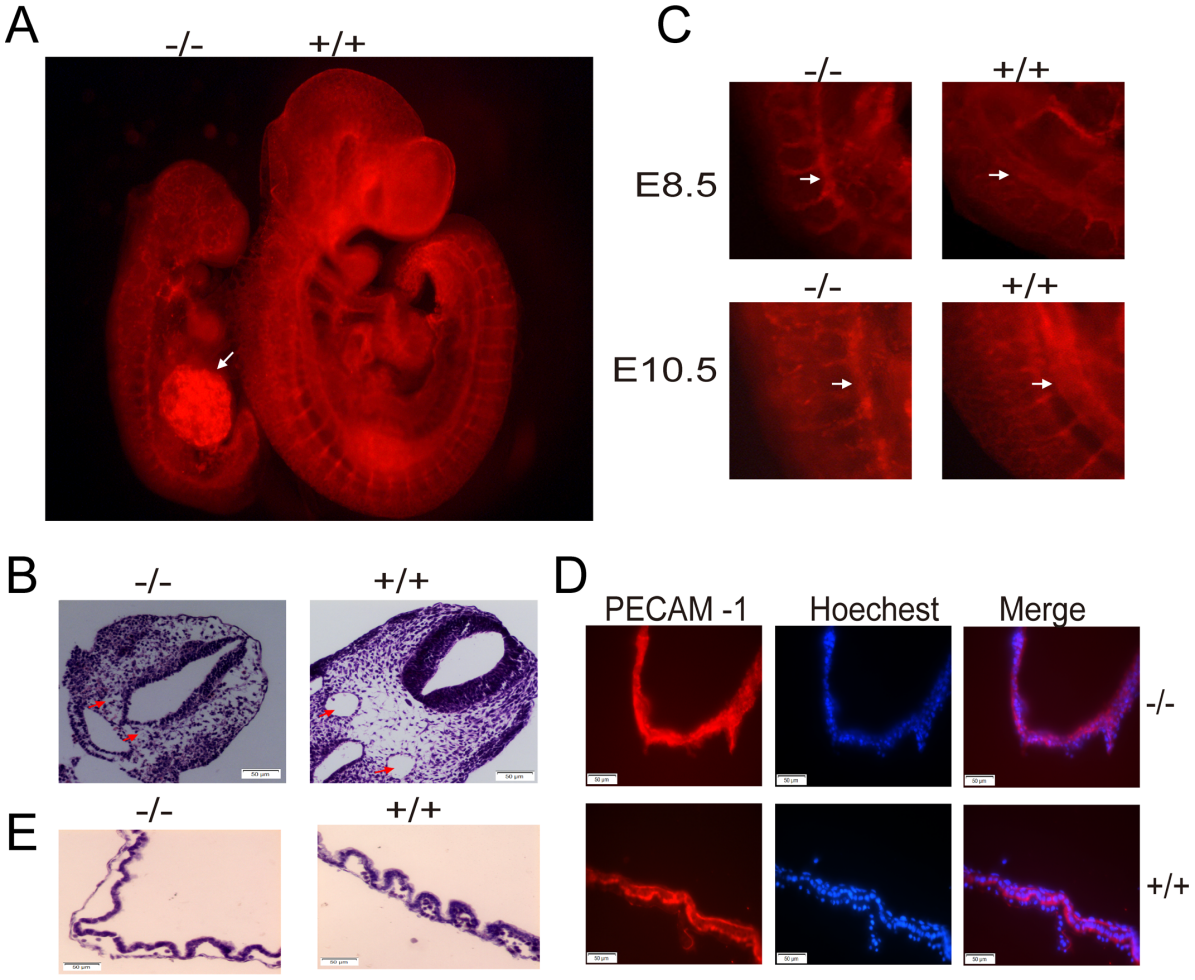
Angiogenesis defects is the direct cause for embryonic lethality in KCTD10^−/−^ embryos. (A), the wild type (+/+) and mutant (−/−) embryos at E10.5 were stained using PECAM-1. (B), HE staining of E10.5 embryos. The red arrow indicates the abnormal dorsal aorta in the KCTD10^−/−^ embryos. (C), wild type (+/+) and mutant (−/−) embryos at E8.5 and E10.5 were stained using PECAM-1, showing the changes of dorsal aorta (DA) development. (D), the yolk sacs at E10.5 were isolated and fixed, frozen sectioned, and immunostained using PECAM-1. The yolk sac showed disorganized in the KCTD10^−/−^ embryos.(E), yolk sacs were dissected from the wild type (+/+) and mutant (−/−) embryos at E10.5 and paraffin sectioned, and then stained using Hemotoxylin/eosin. Enlarged lacunae between the endodermal and mesodermal layers in the mutant (−/−) embryos were observed.

When we isolated E10.5 embryos, we found the vitelline circulation on the yolk sac of KCTD10^−/−^ embryos was absent compared to that in wild type embryos (Fig. 3A). These embryos were severely retarded (Fig. 3B) and the pericardial space was enlarged (white arrows in Fig. 3A), indicating embryonic circulation defects. We then visualized the vascular network of mutant embryos and littermates by staining with an antibody against platelet endothelial cell adhesion molecule-1 (PECAM-1), a specific marker for vascular endothelial cells [26]. In the mutant yolk sac, the primary vascular plexus appeared to form normally, indicating no apparent defects in vasculogenesis in the mutants. But the caliber of the major vitelline arteries and the arterial branching were reduced (Fig. 3C), suggesting that the primary vascular plexus failed to remodel and form blood vessels in the mature yolk sac. In addition, reduced tip cell formation was also observed in the abnormal yolk sac (Fig. 3D). Vascular defects were also seen in the KCTD10^−/−^ embryos, among the 60% of the KCTD10^−/−^ embryos, the umbilical artery (UA) became a big ball that was strongly stained by PECAM-1 (white arrow in Fig. 4A). In all of the KCTD10^−/−^ embryos, the internal carotid artery (ICA), dorsal aorta (DA), arterial branches, and cardinal veins were disorganized and much thinner than those in the wild types. All these findings were confirmed by hematoxylin and eosin (H&E) staining on cross-sections. As shown in Fig. 4B, the dorsal aorta was abnormal or even missing in some severe phenotypes. We compared the dorsal aorta between E8.5 and E10.5 embryos that were immunostained by PECAM-1 (Fig. 4C). At E10.5, the dorsal aorta showed no growth in mutant embryos compared to that in E8.5 embryos (white arrows in Fig. 4C), which further confirmed the conclusion that angiogenesis defects is the direct cause for embryonic lethality. In the PECAM-1 immunostained yolk sac frozen sections, we found that the endothelial cells of the yolk sac were poorly organized in the homozygotes KCTD10^−/−^ compared to those in wild type embryos (Fig. 4D). H&E staining of E10.5 yolk sac sections revealed that the abnormal yolk sac vascular surface was due to the formation of dramatically enlarged endothelial-lined lacunae between the endoderm and mesoderm layers (Fig. 4E).

### KCTD10 deficient embryos show heart defects

**Figure 5:**
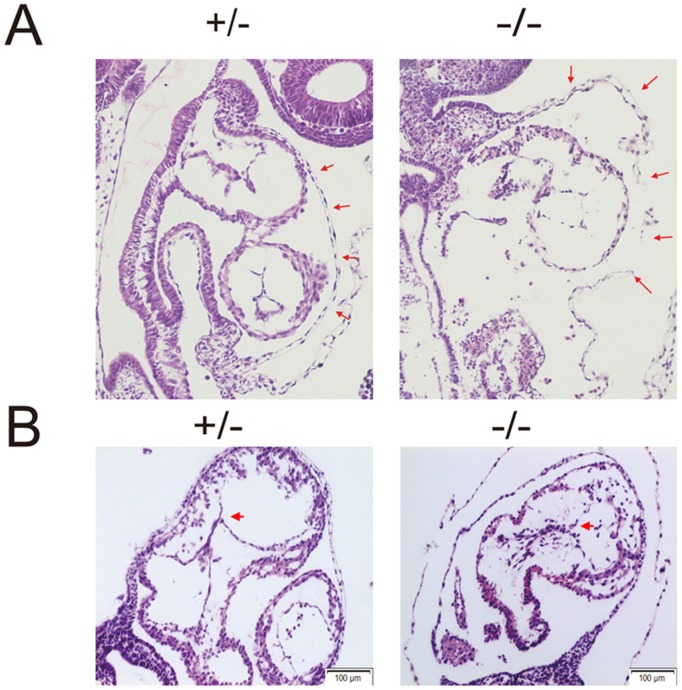
KCTD10-deficient embryos show heart defects. (A), embryos with almost the same somites were cut longitudinally in paraffin, and stained using Hemotoxylin/eosin. The red arrows show the enlarged pericardial edema in mutant embryos. (B), embryos with almost the same somites were cut athwartships in paraffin, and the red arrowheads show the atrioventricular valve defects in the KCTD10^−/−^ embryos.

The disruption of KCTD10 in mice caused heart developmental failure. As described above, the mutant embryos had a dramatically enlarged pericardial edema, which was different from that in wild type littermates (Fig. 3A, B). To characterize the defects in detail, the KCTD10^−/−^ and wild type embryos were sectioned and H&E stained for histological analysis. The mutant embryos showed extended pericardial edema, and the myocardial wall was relatively thinner compared with that in the wild type embryos (Fig. 5A, red arrows). In addition, KCTD10^−/−^ embryos exhibited defects in cardiac valve formation. An obvious atrioventricular valve (AVV) presented in the wild type embryos, but showed defects in the KCTD10^−/−^ embryonic heart (Fig. 5B, red arrow heads). Thus, KCTD10 loss of function leads to heart defects during the early embryonic development.

### KCTD10 is induced by VEGF in a dose- and time-dependent manner

**Figure 6:**
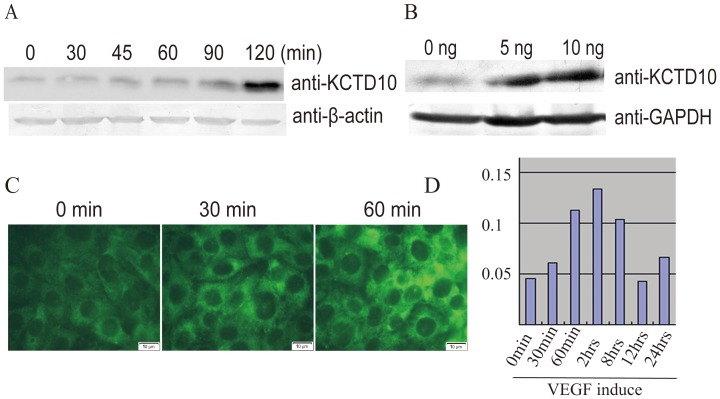
KCTD10 is induced by VEGF in a dose- and time-dependent manner. (A), HUVECs were incubated with 10 ng/mL VEGF for 0 min, 30 min, 45 min, 60 min, 90 min and 120 min, the cell extractions were subjected to SDS-PAGE, and immunoblotted with polyclonal antibody against KCTD10 and β-actin separately. (B), HUVECs were incubated with 0, 5, 10 ng/mL VEGF for 120 min, the cell extractions were subjected to SDS-PAGE, and immune-blotted with polyclonal antibody against KCTD10 and β-actin separately. (C), HUVECs were incubated with 10 ng/mL VEGF for 0 min, 30 min, and 60 min, and the cells were fixed and immune-stained polyclonal antibody against KCTD10. (D), HUVECs were treated with 10 ng/mL VEGF for indicated time, and the total RNA of the cells were extracted, RT-PCR analysis showing the mRNA levels of KCTD10.

Vascular endothelial growth factor (VEGF) plays a critical role in angiogenesis. Most of the factors that affect embryonic vascular maturation are downstream targets of VEGF. Based on the defects in angiogenesis and heart development in KCTD10 deficient embryos, we investigated whether KCTD10 was regulated by VEGF. As described in the Materials and Methods, HUVECs were treated by VEGF-A_165_ at a concentration of 10 ng/mL for different time durations (0, 30, 45, 60, 90, 120 min), the protein levels of KCTD10 were upregulated (Fig. 6A) in a time-dependent manner. As shown in Fig. 6B, the expression of KCTD10 increased when the dose of VEGF was added (0, 5, 10 ng/mL). In order to confirm the results, immunofluorescence staining was performed and enhanced protein levels of KCTD10 were detected (Fig. 6C) after treatment. The total RNA of HUVECs were extracted after 10 ng/mL VEGF treatment for indicated time, RT-PCR analysis showed the mRNA levels of KCTD10 increased(Fig. 6D). These results demonstrated that KCTD10 is time- and dose- dependently regulated by VEGF.

### KCTD10 negatively regulates Notch signaling

**Figure 7:**
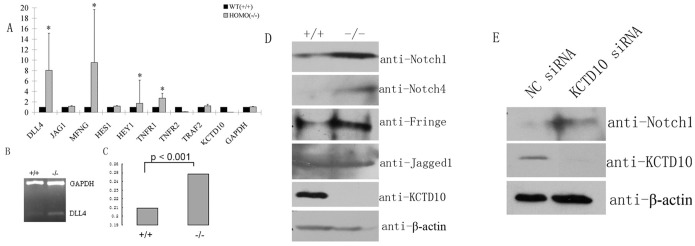
KCTD10 negatively regulates Notch signaling. (A), total RNA was extracted from the mutant embryos that have the same number of somites. Quantitative real-time PCR results showed the mRNA levels of indicated gene in KCTD10^−/−^ embryos and in wild type embryos. (B), Semi-quantitative real-time PCR analysis confirmed the mRNA changes of Dll4. (C), Densitometric analysis of the Dll4/GAPDH ratio in (B). (D), the total proteins were extracted from KCTD10^−/−^ embryos and wild type embryos, western blotting analysis showed the protein changes of Notch signaling in KCTD10^−/−^ embryos. (E), HUVECs were transfected with KCTD10 siRNAs and negative control siRNAs, the cell lysates were subjected to SDS-PAGE and detected by the indicated antibodies.

The phenotype of KCTD10^−/−^ embryos described above were highly similar to that in Dll4^+/−^ mice [27], Notch1/4 mutant mice [15] and Hey1/Hey2 double knockout mice [20]. Therefore, we asked whether the disruption of KCTD10 affects the Notch signaling pathway. The total RNAs of embryos were extracted and quantitative real-time PCR (Q-RT-PCR) analysis were performed. As shown in Fig. 7A, the mRNA levels of Dll4, Fringe, Hey1, and TNFR1 robustly increased in the homozygous KCTD10^−/−^ mouse embryos, whereas the mRNA levels of TNFR2 decreased. The mRNA levels of Jagged1 and Hes1 did not change. Dll4 up-regulation was further confirmed by semi-quantitative reverse transcription PCR (Figs. 7B, C). These data demonstrated that Dll4 is possibly involved in the KCTD10 disruption-mediated vascular defects.

Furthermore, the protein levels of Notch1, Notch4, Fringe, and Jagged1 were measured by western blotting. As shown in Fig. 7D, compared to the wild types, the protein levels of Notch1, Notch4, and Fringe were up-regulated in homozygous KCTD10^−/−^ embryos, while Jagged1 showed no change, which were consistent with the Q-PCR results. In addition, the protein levels of Notch1 and Fringe were also up-regulated in KCTD10 knockdown HUVECs (Fig. 7E). All these data support the hypothesis that the disruption of KCTD10 results in Dll4 up-regulation, followed by the activation of Notch signaling.

### KCTD10 interacts with Notch1 and mediates its proteolytic degradation by Cullin3

**Figure 8:**
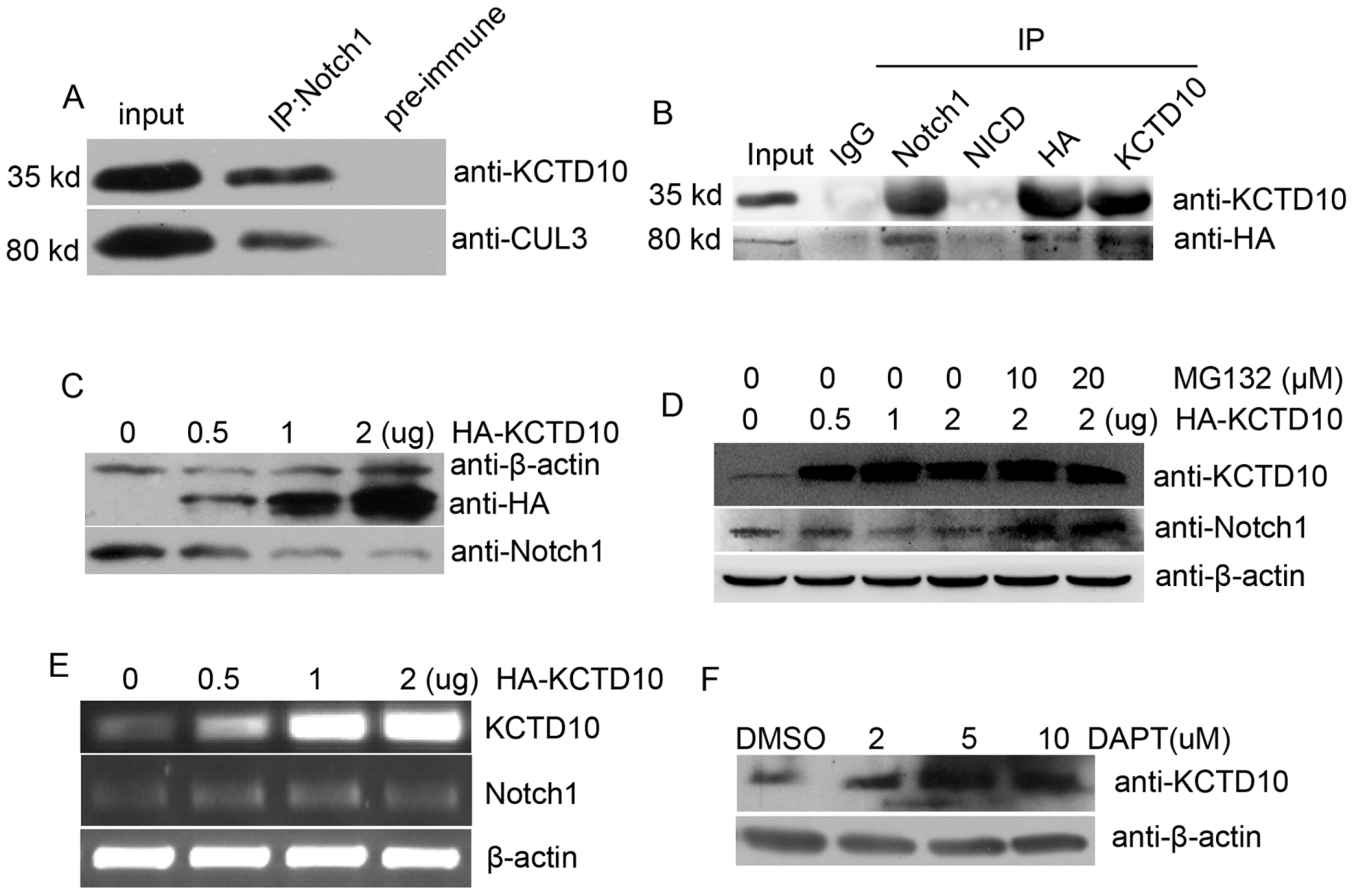
KCTD10 may mediate Notch1 proteolytic degradation by Cullin3. (A), total proteins were extracted from KCTD10^−/−^ embryos and in wild type embryos, endogenous immunoprecipitation was performed to show KCTD10 and cullin3 exist in the immune complex of Notch1 simultaneously. However, rabbit preimmune IgG did not recognize any target protein. (B), HUVECs were transfected with HA-cullin3 and harvested 24 h after transfection, the cell lysate were immunoprecipitated by the antibodis against Notch1, NICD, HA, and KCTD10. The precipitated protein were separated by 10% SDS-PAGE and immunblotted with anti-HA and anti-KCTD10 antibody. (C, D), HUVECs were transfected with an increasing amount of pCMV-HA-KCTD10 (0, 0.5, 1, 2 µg) and treated with MG132 (D), and harvested 8 h after transfection. Cell lysates were prepared and separated using 10% SDS-PAGE electrophoresis. A single blot membrane was cut into strips based on molecular weight and incubated with anti-KCTD10, anti-Notch1 (Santa), and β-actin antibodies to detect the KCTD10, Notch1 and β-actin protein levels, respectively. (E), HUVECs were transfected with an increasing amount of pCMV-HA-KCTD10 (0, 0.5, 1, 2 µg), the total RNA were extracted and the mRNA levels of KCTD10 and Notch1 were detected. (F), HUVECs were treated by increasing amount of DAPT for 2 h, and the KCTD10 and β-actin protein levels were detected using Western blotting.

As KCTD10 contains a conserved BTB/POZ domain and a potassium channel tetramerisation (K-tetra) domain (a relative of BTB/POZ domain) at the N-terminus. The BTB domain was known to be a highly conserved protein-protein interaction motif in multiple species. BTB-domain-containing proteins have been reported to act as substrate-specific adaptors for multimeric cullin3 ligase reactions by recruiting proteins for ubiquitination and mediating subsequent degradation of the substrates [28]–[32]. We then asked whether KCTD10 induces the Notch degradation. In our study, HUVEC lysates were immunoprecipitated by rabbit polyclonal antibody against Notch1 or a negative control IgG, and the immunoprecipitates were detected by mouse monoclonal anti-cullin3 and polyclonal anti-KCTD10 antibodies, respectively. As shown in Fig. 8A, KCTD10 and cullin3 exist simultaneously in the Notch1 immune complex, but the preimmune IgG did not show any reactivity in blots. To further confirm the results, HUVECs were transfected with HA-cullin3, and the cell lysates were immunoprecipitated by antibodies against Notch1, NICD, HA, and KCTD10, immunoblotted by anti-HA and anti-KCTD10 antibody, Notch1 existed in the immune complexes of HA-cullin3 and KCTD10 and KCTD10 existed in the immune complexes of HA-cullin3. But in the immune complex of NICD (intracellular domains of Notch1), we failed to detect either KCTD10 or cullin3 (Fig. 8B). These results further confirmed the interaction between Notch1, KCTD10 and cullin3. We next wondered whether Notch1 proteins levels were affected by KCTD10. To this end, HUVECs were transfected with increasing amounts of pCMV-HA-KCTD10 (0, 0.5, 1, 2 µg). Western blotting results showed that the endogenous Notch1 protein decreased while the amounts of pCMV-HA-KCTD10 increased (Fig. 8C, D), suggesting that KCTD10 disrupted the protein stability of Notch1. Furthermore, MG132 treatment attenuated the changes(Fig. 8D), and the mRNA levels of Notch1 did not affected by KCTD10 (Fig. 8E), indicating proteolytic degradation of Notch1by KCTD10. We then blocked Notch signaling activation by DAPT, which is a γ-secretase inhibitor in HUVECs [27]. Western blotting analysis showed that the KCTD10 protein levels increased as the DAPT concentration increased (Fig. 8F), suggesting that KCTD10 mediated the γ-secretase of proteolytic processing of Notch1.

In summary, KCTD10 interacts with Notch1 and Cullin3 simultaneously, and decreases the Notch1 protein stability, which indicates that KCTD10 may regulate Notch1 proteolytic degradation by interacting with cullin3.

## Discussion

Recently, there are publications showed that KCTD10 plays critical roles for heart development in zebrafish [33], [34]. Here we explored whether KCTD10 plays critical roles in mammalian development. Like all other members in PDIP1 gene family, KCTD10 contains a BTB/POZ domain at the N-terminals, which is a highly conserved protein-protein interacting domain found in many essential transcription regulators that are involved in various developmental processes, and is important for homeostasis, cell differentiation, and even oncogenesis [35]–[37]. The BTB domain is the key functional domain that directly inhibits endothelial cell tube formation and reduces VEGF expression, another BTB domain protein, PLZF, was reported to inhibit endothelial cell angiogenesis in HUVECs [38]. VEGF is known as the most critical molecule controlling blood vessel morphogenesis. In our study, western blotting, RT-PCR and immunofluorescence showed that KCTD10 is VEGF-inducible in a time- and dose-dependent manner, suggesting KCTD10 might be regulated by a VEGF feed-back loop. Notch signaling is known to be the downstream target of VEGF, and Dll4 expression is induced in response to VEGF [39]. Dll4 is the specific mammalian endothelial ligand for autocrine endothelial Notch signaling, and is required in a dosage-sensitive manner for normal arterial patterning in development [27]. Dll4 inhibits the angiogenic response of adjacent ECs to VEGF stimulation by mediating Notch signaling. Reduction of the Dll4 protein level or blocking of Notch signaling blocking enhances the formation of tip cells, resulting in dramatically increased sprouting, branching and fusion of endothelial tubes [27], [39]–[43]. Heterozygous deletion of Dll4 results in prominent albeit variable defects in artery, and the down-regulation of Notch downstream target genes [44]. In our study, we deleted the 2^nd^ exon of mouse KCTD10, which destroyed the BTB domain and caused loss of function of this gene. A homozygous deletion of KCTD10 in mice caused embryonic lethality because of cardiovascular defects, accompanied by elevated Dll4 levels and activated Fringe, Hey1, and TNFR1 in Notch signaling. These results are consistent with previous research that KCTD10 is TNF-α inducible [4], but the detailed mechanisms need to be further explored.

The Notch signaling pathway is a critical component of vascular formation and morphogenesis in both development and pathology. It was reported that Notch was required for endocardial differentiation and formation of the primordial cardiac valve during the cardiac valve development [45], [46]. In a normal embryo, Notch signaling induces endocardial expression of the transcriptional repressor Snail, and in turn, represses VE-cadherin expression, allowing the epithelial-to-mesenchyme transformation (EMT), followed by the formation of the valvular primordium [46]. Abrogation of Notch signaling in mouse or zebrafish blocks EMT [47]. The Notch downstream target gene Hey was shown to control cardiomyocyte differentiation and EMT in endocardial cells [48]. It is possible that deletion of KCTD10 increases Notch1 and then up-regulates Hey1, resulting in a thinner myocardium and EMT defects in the homozygous KCTD10^−/−^ embryos.

Notch receptors are expressed on the cell surface as heterodimeric proteins. They are composed of an extracellular domain containing up to 36 EGF-like repeats followed by 3 cysteine rich LIN repeats and an intracellular domain containing multiple protein-protein interaction domains. Notch signaling is triggered upon ligand-receptor interaction, which induces two sequential proteolytic cleavages. The first cleavage is in the extracellular domain, and is mediated by metalloproteases of the ADAM family; the second cleavage is within the transmembrane domain, and it is mediated by presenilin (PS) γ-secretase activity. The secondary cleavage allows the release and translocation of Notch intracellular domain (NICD) into the nucleus [49]. Consistent with reports that the KCTD10 BTB/POZ domain mediates protein-protein interactions and is involved in proteolysis mediated by Cullin3 [11], [31], [50]–[52], we revealed that KCTD10 interacts with Cullin3 and Notch1 simultaneously, and the γ-secretase-specific inhibitor DAPT [27] regulates the KCTD10 protein level. The possible mechanism is that KCTD10 mediates the second proteolytic cleavage and transfers the NICD into the nucleus. Thus, KCTD10 deletion causes Notch1 accumulation, which stimulated more Dll4, and leads to disruption of angiogenesis.

In summary, we generated KCTD10 knockout mice by disrupting exon 2, leading to loss of function of KCTD10. This study provided evidence that KCTD10 plays important roles in embryonic angiogenesis during mammalian development, cardiovascular development, and negative regulation of Notch signaling. Thus, KCTD10 is one of the most important factors in regulating embryonic development and angiogenesis.

## References

[1] He H, Tan CK, Downey KM, So AG (2001) A tumor necrosis factor alpha- and interleukin 6-inducible protein that interacts with the small subunit of DNA polymerase delta and proliferating cell nuclear antigen. Proc Natl Acad Sci U S A 98: 11979–11984. 11593007 10.1073/pnas.221452098 PMC59753

[2] Zhou J, Hu X, Xiong X, Liu X, Liu Y, et al (2005) Cloning of two rat PDIP1 related genes and their interactions with proliferating cell nuclear antigen. J Exp Zool A Comp Exp Biol 303: 227–240. 15726626 10.1002/jez.a.150

[3] Zhou J, Ren K, Liu X, Xiong X, Hu X, et al (2005) A novel PDIP1-related protein, KCTD10, that interacts with proliferating cell nuclear antigen and DNA polymerase delta. Biochim Biophys Acta 1729: 200–203. 15982757 10.1016/j.bbaexp.2005.05.005

[4] Zhou J, Fan C, Zhong Y, Liu Y, Liu M, et al (2005) Genomic organization, promoter characterization and roles of Sp1 and AP-2 in the basal transcription of mouse PDIP1 gene. FEBS Lett 579: 1715–1722. 15757666 10.1016/j.febslet.2005.02.027

[5] Liu R, Zhou A, Ren D, He A, Hu X, et al (2009) Transcription factor specificity protein 1 (SP1) and activating protein 2alpha (AP-2alpha) regulate expression of human KCTD10 gene by binding to proximal region of promoter. FEBS J 276: 1114–1124. 19154347 10.1111/j.1742-4658.2008.06855.x

[6] Wang Y, Zheng Y, Luo F, Fan X, Chen J, et al (2009) KCTD10 interacts with proliferating cell nuclear antigen and its down-regulation could inhibit cell proliferation. J Cell Biochem 106: 409–413. 19125419 10.1002/jcb.22026

[7] Kubota D, Yoshida A, Tsuda H, Suehara Y, Okubo T, et al (2013) Gene expression network analysis of ETV1 reveals KCTD10 as a novel prognostic biomarker in gastrointestinal stromal tumor. PLoS One 8: e73896. 23977394 10.1371/journal.pone.0073896 PMC3747077

[8] Sun JK, Zhang B, Zhang J, Zhou JL (2007) [Preparation of mouse KCTD10 antibody and expression analysis of KCTD10 in neuroepithelium of neural tube and dorsal root ganglion]. Sheng Wu Gong Cheng Xue Bao 23: 1011–1016. 18257228

[9] Carmeliet P, Ferreira V, Breier G, Pollefeyt S, Kieckens L, et al (1996) Abnormal blood vessel development and lethality in embryos lacking a single VEGF allele. Nature 380: 435–439. 8602241 10.1038/380435a0

[10] Gale NW, Yancopoulos GD (1999) Growth factors acting via endothelial cell-specific receptor tyrosine kinases: VEGFs, angiopoietins, and ephrins in vascular development. Genes Dev 13: 1055–1066. 10323857 10.1101/gad.13.9.1055

[11] Sekhar KR, Rachakonda G, Freeman ML (2010) Cysteine-based regulation of the CUL3 adaptor protein Keap1. Toxicol Appl Pharmacol 244: 21–26. 19560482 10.1016/j.taap.2009.06.016 PMC2837771

[12] Zhou W, Wang G, Guo S (2013) Regulation of angiogenesis via Notch signaling in breast cancer and cancer stem cells. Biochim Biophys Acta. 10.1016/j.bbcan.2013.10.003 PMC7983532 24183943

[13] Shawber CJ, Kitajewski J (2004) Notch function in the vasculature: insights from zebrafish, mouse and man. Bioessays 26: 225–234. 14988924 10.1002/bies.20004

[14] Swiatek PJ, Lindsell CE, del Amo FF, Weinmaster G, Gridley T (1994) Notch1 is essential for postimplantation development in mice. Genes Dev 8: 707–719. 7926761 10.1101/gad.8.6.707

[15] Krebs LT, Xue Y, Norton CR, Shutter JR, Maguire M, et al (2000) Notch signaling is essential for vascular morphogenesis in mice. Genes Dev 14: 1343–1352. 10837027 PMC316662

[16] Thurston G, Gale NW (2004) Vascular endothelial growth factor and other signaling pathways in developmental and pathologic angiogenesis. Int J Hematol 80: 7–20. 15293563 10.1532/ijh97.04065

[17] McCright B, Gao X, Shen L, Lozier J, Lan Y, et al (2001) Defects in development of the kidney, heart and eye vasculature in mice homozygous for a hypomorphic Notch2 mutation. Development 128: 491–502. 11171333 10.1242/dev.128.4.491

[18] Delibas S, Guven H, Comoglu SS (2009) A case report about CADASlL: mutation in the NOTCH 3 receptor. Acta Neurol Taiwan 18: 262–266. 20329594

[19] Iso T, Kedes L, Hamamori Y (2003) HES and HERP families: multiple effectors of the Notch signaling pathway. J Cell Physiol 194: 237–255. 12548545 10.1002/jcp.10208

[20] Fischer A, Schumacher N, Maier M, Sendtner M, Gessler M (2004) The Notch target genes Hey1 and Hey2 are required for embryonic vascular development. Genes Dev 18: 901–911. 15107403 10.1101/gad.291004 PMC395849

[21] Liu P, Jenkins NA, Copeland NG (2003) A highly efficient recombineering-based method for generating conditional knockout mutations. Genome Res 13: 476–484. 12618378 10.1101/gr.749203 PMC430283

[22] Nagy A, Rossant J, Nagy R, Abramow-Newerly W, Roder JC (1993) Derivation of completely cell culture-derived mice from early-passage embryonic stem cells. Proc Natl Acad Sci U S A 90: 8424–8428. 8378314 10.1073/pnas.90.18.8424 PMC47369

[23] Farley FW, Soriano P, Steffen LS, Dymecki SM (2000) Widespread recombinase expression using FLPeR (flipper) mice. Genesis 28: 106–110. 11105051

[24] Lakso M, Pichel JG, Gorman JR, Sauer B, Okamoto Y, et al (1996) Efficient in vivo manipulation of mouse genomic sequences at the zygote stage. Proc Natl Acad Sci U S A 93: 5860–5865. 8650183 10.1073/pnas.93.12.5860 PMC39152

[25] Henrique D, Adam J, Myat A, Chitnis A, Lewis J, et al (1995) Expression of a Delta homologue in prospective neurons in the chick. Nature 375: 787–790. 7596411 10.1038/375787a0

[26] Baldwin HS, Shen HM, Yan HC, DeLisser HM, Chung A, et al (1994) Platelet endothelial cell adhesion molecule-1 (PECAM-1/CD31): alternatively spliced, functionally distinct isoforms expressed during mammalian cardiovascular development. Development 120: 2539–2553. 7956830 10.1242/dev.120.9.2539

[27] Suchting S, Freitas C, le Noble F, Benedito R, Breant C, et al (2007) The Notch ligand Delta-like 4 negatively regulates endothelial tip cell formation and vessel branching. Proc Natl Acad Sci U S A 104: 3225–3230. 17296941 10.1073/pnas.0611177104 PMC1805603

[28] Bayon Y, Trinidad AG, de la Puerta ML, Del Carmen Rodriguez M, Bogetz J, et al (2008) KCTD5, a putative substrate adaptor for cullin3 ubiquitin ligases. FEBS J 275: 3900–3910. 18573101 10.1111/j.1742-4658.2008.06537.x

[29] Furukawa M, He YJ, Borchers C, Xiong Y (2003) Targeting of protein ubiquitination by BTB-Cullin 3-Roc1 ubiquitin ligases. Nat Cell Biol 5: 1001–1007. 14528312 10.1038/ncb1056

[30] Geyer R, Wee S, Anderson S, Yates J, Wolf DA (2003) BTB/POZ domain proteins are putative substrate adaptors for cullin 3 ubiquitin ligases. Mol Cell 12: 783–790. 14527422 10.1016/s1097-2765(03)00341-1

[31] Pintard L, Willems A, Peter M (2004) Cullin-based ubiquitin ligases: Cul3-BTB complexes join the family. EMBO J 23: 1681–1687. 15071497 10.1038/sj.emboj.7600186 PMC394240

[32] Xu L, Wei Y, Reboul J, Vaglio P, Shin TH, et al (2003) BTB proteins are substrate-specific adaptors in an SCF-like modular ubiquitin ligase containing CUL-3. Nature 425: 316–321. 13679922 10.1038/nature01985

[33] Hu X, Gan S, Xie G, Li L, Chen C, et al. (2014) KCTD10 is critical for heart and blood vessel development of zebrafish. Acta Biochim Biophys Sin (Shanghai). 10.1093/abbs/gmu017 24705121

[34] Tong X, Zu Y, Li Z, Li W, Ying L, et al (2014) Kctd10 regulates heart morphogenesis by repressing the transcriptional activity of Tbx5a in zebrafish. Nat Commun 5: 3153. 24430697 10.1038/ncomms4153

[35] Chang CC, Ye BH, Chaganti RS, Dalla-Favera R (1996) BCL-6, a POZ/zinc-finger protein, is a sequence-specific transcriptional repressor. Proc Natl Acad Sci U S A 93: 6947–6952. 8692924 10.1073/pnas.93.14.6947 PMC38914

[36] Shaffer AL, Yu X, He Y, Boldrick J, Chan EP, et al (2000) BCL-6 represses genes that function in lymphocyte differentiation, inflammation, and cell cycle control. Immunity 13: 199–212. 10981963 10.1016/s1074-7613(00)00020-0

[37] Zollman S, Godt D, Prive GG, Couderc JL, Laski FA (1994) The BTB domain, found primarily in zinc finger proteins, defines an evolutionarily conserved family that includes several developmentally regulated genes in Drosophila. Proc Natl Acad Sci U S A 91: 10717–10721. 7938017 10.1073/pnas.91.22.10717 PMC45093

[38] Rho SB, Choi K, Park K, Lee JH (2010) Inhibition of angiogenesis by the BTB domain of promyelocytic leukemia zinc finger protein. Cancer Lett 294: 49–56. 20236758 10.1016/j.canlet.2010.01.021

[39] Lobov IB, Renard RA, Papadopoulos N, Gale NW, Thurston G, et al (2007) Delta-like ligand 4 (Dll4) is induced by VEGF as a negative regulator of angiogenic sprouting. Proc Natl Acad Sci U S A 104: 3219–3224. 17296940 10.1073/pnas.0611206104 PMC1805530

[40] Noguera-Troise I, Daly C, Papadopoulos NJ, Coetzee S, Boland P, et al (2006) Blockade of Dll4 inhibits tumour growth by promoting non-productive angiogenesis. Nature 444: 1032–1037. 17183313 10.1038/nature05355

[41] Sainson RC, Aoto J, Nakatsu MN, Holderfield M, Conn E, et al (2005) Cell-autonomous notch signaling regulates endothelial cell branching and proliferation during vascular tubulogenesis. FASEB J 19: 1027–1029. 15774577 10.1096/fj.04-3172fje

[42] Hellstrom M, Phng LK, Hofmann JJ, Wallgard E, Coultas L, et al (2007) Dll4 signalling through Notch1 regulates formation of tip cells during angiogenesis. Nature 445: 776–780. 17259973 10.1038/nature05571

[43] Ridgway J, Zhang G, Wu Y, Stawicki S, Liang WC, et al (2006) Inhibition of Dll4 signalling inhibits tumour growth by deregulating angiogenesis. Nature 444: 1083–1087. 17183323 10.1038/nature05313

[44] Gale NW, Dominguez MG, Noguera I, Pan L, Hughes V, et al (2004) Haploinsufficiency of delta-like 4 ligand results in embryonic lethality due to major defects in arterial and vascular development. Proc Natl Acad Sci U S A 101: 15949–15954. 15520367 10.1073/pnas.0407290101 PMC524697

[45] Jain R, Rentschler S, Epstein JA (2010) Notch and cardiac outflow tract development. Ann N Y Acad Sci 1188: 184–190. 20201902 10.1111/j.1749-6632.2009.05099.x PMC2975619

[46] Timmerman LA, Grego-Bessa J, Raya A, Bertran E, Perez-Pomares JM, et al (2004) Notch promotes epithelial-mesenchymal transition during cardiac development and oncogenic transformation. Genes Dev 18: 99–115. 14701881 10.1101/gad.276304 PMC314285

[47] Chaffer CL, Thompson EW, Williams ED (2007) Mesenchymal to epithelial transition in development and disease. Cells Tissues Organs 185: 7–19. 17587803 10.1159/000101298

[48] Kokubo H, Tomita-Miyagawa S, Hamada Y, Saga Y (2007) Hesr1 and Hesr2 regulate atrioventricular boundary formation in the developing heart through the repression of Tbx2. Development 134: 747–755. 17259303 10.1242/dev.02777

[49] Boucher J, Gridley T, Liaw L (2012) Molecular pathways of notch signaling in vascular smooth muscle cells. Front Physiol 3: 81. 22509166 10.3389/fphys.2012.00081 PMC3321637

[50] Marshall J, Blair LA, Singer JD (2011) BTB-Kelch proteins and ubiquitination of kainate receptors. Adv Exp Med Biol 717: 115–125. 21713671 10.1007/978-1-4419-9557-5_10 PMC3929045

[51] Willems AR, Schwab M, Tyers M (2004) A hitchhiker’s guide to the cullin ubiquitin ligases: SCF and its kin. Biochim Biophys Acta 1695: 133–170. 15571813 10.1016/j.bbamcr.2004.09.027

[52] Jiang J (2006) Regulation of Hh/Gli signaling by dual ubiquitin pathways. Cell Cycle 5: 2457–2463. 17102630 10.4161/cc.5.21.3406

